# Force Transmission in Disordered Fibre Networks

**DOI:** 10.3389/fcell.2022.931776

**Published:** 2022-06-30

**Authors:** José Ruiz-Franco, Jasper van Der Gucht

**Affiliations:** Laboratory of Physical Chemistry and Soft Matter, Wageningen University and Research, Wageningen, Netherlands

**Keywords:** fiber networks, local stresses, force transmission, connectivity, bending rigidity, graph theory

## Abstract

Cells residing in living tissues apply forces to their immediate surroundings to promote the restructuration of the extracellular matrix fibres and to transmit mechanical signals to other cells. Here we use a minimalist model to study how these forces, applied locally by cell contraction, propagate through the fibrous network in the extracellular matrix. In particular, we characterize how the transmission of forces is influenced by the connectivity of the network and by the bending rigidity of the fibers. For highly connected fiber networks the stresses spread out isotropically around the cell over a distance that first increases with increasing contraction of the cell and then saturates at a characteristic length. For lower connectivity, however, the stress pattern is highly asymmetric and is characterised by force chains that can transmit stresses over very long distances. We hope that our analysis of force transmission in fibrous networks can provide a new avenue for future studies on how the mechanical feedback between the cell and the ECM is coupled with the microscopic environment around the cells.

## 1 Introduction

Living tissues are constituted by the extracellular matrix (ECM), a complex network of proteins and polysaccharides that gives structural support to surrounding cells. In animal tissues, the main component of the ECM is collagen, which forms a crosslinked network of stiff fibres that provides the ECM with its elasticity and mechanical strength (Mouw et al., 2014; Burla et al., 2019a). Cells are embedded within this network and are linked to the matrix by focal adhesion complexes (FAs), which act as physical anchors *via* which cells can mechanically interact with their environment (Totsukawa et al., 2004; Lecuit et al., 2011). Indeed, many cellular processes are regulated by mechanical feedback between cells and the ECM. Cells actively exert forces on the surrounding matrix, leading to structural reorganisations in the surrounding network, like fibre alignment, plastic rearrangements, and densification around the cell (Vader et al., 2009; Kim et al., 2017; Sopher et al., 2018; Goren et al., 2020). Cells also sense the mechanical properties of the surrounding medium. For example, cancer cells exhibit a preferential migration to regions with higher stiffness (durotaxis) (Lo et al., 2000; DuChez et al., 2019; Rens and Merks, 2020), and can adapt their shape as a function of the matrix stiffness (Koch et al., 2012). Likewise, wound healing requires contractile forces applied by myofibroblasts around the injured zone (Li and Wang, 2011). Cells also use mechanical signals to communicate with other cells; they actively exert forces on the surrounding matrix, transmitted through the ECM to distant cells (Reinhart-King et al., 2008; Winer et al., 2009; Han et al., 2018). This mechanical signalling is believed to play an essential role in tissue development, as well as in the development of cancer and other diseases (Bates et al., 2007; Hinz et al., 2012). Therefore, understanding how forces propagate in the extracellular matrix is relevant for obtaining fundamental knowledge about biological processes in both healthy and pathological tissue. Likewise, this insight can be revealing in tissue engineering, where tailoring the mechanical properties and cell-matrix interactions are crucial for the successful development of artificial tissues and organs (Chen et al., 2004; Causa et al., 2007; Wegst et al., 2015).

The challenge in describing mechanical signal propagation through the ECM is that the ECM is a very heterogeneous fibre network, with a typical mesh size that is comparable to the size of the cell. This means that continuum theories cannot be used (Notbohm et al., 2015; Ronceray et al., 2016; Han et al., 2018). The heterogeneity of the fibre network is regulated by the network connectivity *z* and the bending rigidity of the fibres, which thereby influence the mechanical response of the ECM. It is well-known that networks with only central-force interactions become mechanically stable only when the connectivity exceeds a critical threshold known as the isostatic point, which has been shown by Maxwell to be equal to *z*_*c*_ = 2*d*, with *d* the spatial dimensionality (Maxwell, 1864). However, the extracellular fibre networks surrounding cells have a lower connectivity, ranging from *z* = 3 for branched networks to *z* = 4 for cross-linked fibres. In particular, collagen networks exhibit an average connectivity $\left\langle z\right\rangle \approx 3.4$ , making them sub-isostatic (Jansen et al., 2018). For such networks, the bending rigidity of the fibres *κ* emerges as an additional mechanism to induce network stability (Broedersz et al., 2011). The bending rigidity is related to the persistence length *l*_*p*_ of the fibres as $l_{p}=\kappa /\left(k_{B}T\right)$ , which describes the length scale of undulations of a polymer driven by thermal energy *k*_*B*_ *T*. For collagen fibres the persistence length is typically much larger than the contour length of the fibres, which means that collagen fibres are stiff and entropic effects due to fluctuations can be neglected. The interplay between connectivity and fibre bending leads to a strongly nonlinear mechanical response to applied stresses (Licup et al., 2015; Sharma et al., 2016a; Jansen et al., 2018). At low strains, the network is soft with a response governed by fibre bending and non-affine network reorganisations. At higher strains, alignment of the fibres in the strain direction leads to fibre stretching, making the network much more rigid (Narmoneva et al., 1999; Vader et al., 2009; Broedersz et al., 2011; Licup et al., 2015). It has been shown that this nonlinearity has a pronounced impact on how forces propagate in the network (Baker and Chen, 2012; Jones et al., 2015; Han et al., 2018).

To understand mechanical signalling between cells in the ECM, it is thus necessary to develop a model for force propagation that incorporates the disordered network structure and its mechanical nonlinearity. To do this, we employ a minimalist model based on two-dimensional triangular athermal networks, where the disorder is induced by controlling the connectivity. Such network models have been shown to give a very accurate description of the mechanics of collagen networks (Licup et al., 2015; Sharma et al., 2016a; Burla et al., 2020). To model an embedded cell, we incorporate a rigid circular body, which shrinks in area, generating local compression. We then examine how forces propagate from the contracting cell through the network, using concepts from network theory. Our findings reveal that the propagation in the case of high connectivity is isotropic and limited when the surrounding network around the cell is highly stressed. By contrast, asymmetry emerges at low connectivity, and the transmission achieves larger distances. The bending rigidity in this regime has a more pronounced role in controlling the force transmission.

## 2 Modeling

We perform numerical simulations on 2D diluted triangular networks of *N* × *N* nodes, with *N* = 100, and with spacing *l*_0_. Periodic boundary conditions are applied in all directions. We dilute the lattice by randomly removing bonds with probability 1 − *p*, and remove all dangling ends. This leads to an average network connectivity of $\left\langle z\right\rangle =pz_{max}$ , with *z*_*max*_ = 6 for our triangular lattice.

We model fibrous biopolymers such as collagen by considering stretching and bending rigidity. Thus, we consider every bond in the diluted network as a Hookean spring with stretching modulus *μ*, while sequences of contiguous colinear bonds have an associated bending rigidity *κ*. The Hamiltonian $H=H_{\text{stretch}}+H_{\text{bend}}$ that quantifies the network energy is expressed as

$$H=\frac{\mu }{2}\underset{\left\langle ij\right\rangle }{\sum }\frac{{\left(l_{ij}-l_{ij,0}\right)}^{2}}{l_{ij,0}}+\frac{\kappa }{2}\underset{\left\langle ijk\right\rangle }{\sum }\frac{{\left(\theta_{\mathrm{ijk}}-\theta_{\mathrm{ijk,0}}\right)}^{2}}{l_{\mathrm{ijk,0}}},$$ (1)

where, in the first term, the sum runs over the bonded pairs $\left\langle ij\right\rangle$ , *l*_*ij*_ denotes the distance between the two nodes, and *l*_*ij*,0_ indicates the rest length. The second term accounts for the bending energy and takes the bonded triplets $\left\langle ijk\right\rangle$ , with *θ*_*ijk*_ the angle between the triplet and *θ*_*ijk,0*_ the rest angle, and *l*_*ijk,0*_ = (*l*_*ij*,0_ + *l*_*jk*,0_)/2. We fix *μ* = 1 and *l*_0_ = 1, and define a reduced bending rigidity $\tilde{\kappa }=\kappa /\left(\mu l_{0}^{2}\right)$ to specify the relative importance of bending stiffness compared to stretching stiffness. Since biological fibres are typically much softer with respect to bending than to stretching, we will consider only cases with $\tilde{\kappa }\ll 1$ .

**FIGURE 1:**
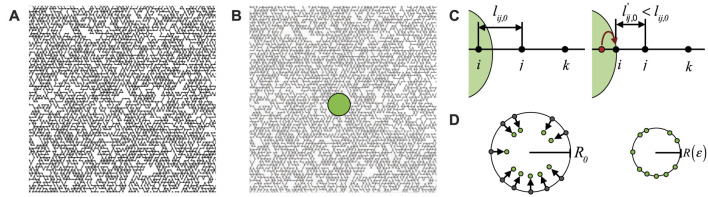
**(A)** Diluted triangular network with *p* = 0.55. **(B)** A circular model cell with radius *R*_0_ = 3*l*_0_ is placed in the center of the network **(C)** All bonds inside the cell are removed, while bonds that cross the cell surface are adjusted by moving the connected nodes at the cell interior to the cell surface, and adjusting the corresponding bond length *l*_*ij*,0_. **(D)** Schematic showing the affine compression of the cell, by moving the nodes on the cell surface inward.

According to Maxwell’s rigidity criterion, the isostatic point (i.e., the connectivity below which the rigidity of the network vanishes) for these networks in the absence of bending stiffness (so for $\tilde{\kappa }=0$ ) is equal to 0.65, while for non-zero $\tilde{\kappa }$ the rigidity threshold is lower, at *p* = 0.442 (Maxwell, 1864; Broedersz et al., 2011). It has been shown that for *p* > 0.65, the mechanical properties of these networks are dominated by stretching of the fibres, while for 0.45 < *p* < 0.65 non-affine fibre bending modes govern the mechanics (Ronceray et al., 2016; Broedersz et al., 2011), Here, we compare four different connectivities, namely *p* = 0.85, 0.75, 0.65, and 0.55, which translates into $\left\langle z\right\rangle =5.1$ , 4.5, 3.9, and 3.3, respectively. In Figure 1A we show an example of the final network for *p* = 0.55. We choose these parameter values to explore a range that includes the connectivity of *in-vitro* reconstituted collagen networks, reported to be in the range 0.5 − 0.65 (Jansen et al., 2018; Burla et al., 2020). The normalized bending rigidity of collagen fibres has been reported to be on the order of 10^−4^, but it can reach higher values for strongly bundled fibres (Jansen et al., 2018; Burla et al., 2020). We therefore explore $\tilde{\kappa }=10^{-4}$ , 10^−3^, and 10^−2^. We furthermore emphasise that the network model that we use here has been shown previously to accurately describe the mechanical properties and fracture of real collagen networks (Broedersz and MacKintosh, 2014; Burla et al., 2020; Tauber et al., 2022).

We then introduce a circular rigid body with radius *R*_0_ = 3*l*_0_ that mimics an embedded cell in the center of the network, as we show in Figure 1B, and we place nodes on the intersection points between the network and the surface of the cell, while adjusting the corresponding equilibrium bond lengths, see Figure 1C. Nodes at the interior of the cell are removed.

Finally, we induce an isotropic contraction of the cell body by applying affine deformation on nodes on the cell surface towards the cell center, as we schematically represent in Figure 1D. This local deformation is quantified by the strain $\epsilon =-\left(R-R_{0}\right)/R_{0}$ , where *R* is the cell’s radius after contraction. After each strain step, fixed to be Δ*ϵ* = 0.001, the network is equilibrated by a minimization of energy using the FIRE algorithm (Bitzek et al., 2006) on the remaining nodes of the network, and with a tolerance *F*_*RMS*_ = 10^−8^. Hence, thermal fluctuations are ignored and the fibre network is modelled as an athermal elastic network. Previous work has shown that this is a good assumption for collagen networks Broedersz et al. (2011), Licup et al. (2015), Rens et al. (2016), Arzash et al. (2020), Burla et al. (2020). The different observables discussed below are averaged over 20 independent simulations for *p* = 0.85 and 0.75, and over 50 for *p* = 0.65 and 0.55, for every $\tilde{\kappa }$ .

## 3 Results

### 3.1 Local Deformation in the Network

**FIGURE 2:**
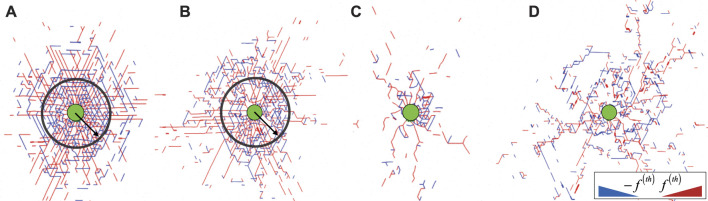
Snapshots for networks with **(A)** *p* = 0.85, **(B)** *p* = 0.75, **(C)** *p* = 0.65 and **(D)** *p* = 0.55, showing all compressed ( $f_{ij}<-f^{\left(th\right)}$ , blue) and stretched ( $f_{ij}>f^{\left(th\right)}$ , red) bonds for $\tilde{\kappa }=10^{-4}$ at *ϵ* = 0.50. Here, the black circle has a radius r* ∼ 10*l*_0_, corresponding to the boundary between the dense and diffuse region observed by computing $\phi \left(r\right)$ . The radial stress in the inner region decays as $\sigma_{rr}\left(r\right)\propto r^{-2}$ . Bond thickness is proportional to force magnitude.

**FIGURE 3:**
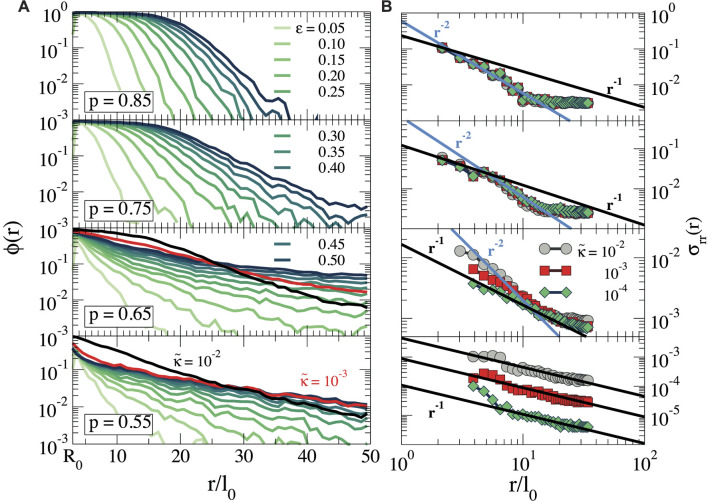
**(A)** Fraction of nodes at a distance *r*, that is, stretched or compressed, $\phi \left(r\right)$ , in semi-log scale, for $\tilde{\kappa }=10^{-4}$ and for different strains *ϵ*. For *p* = 0.65 and *p* = 0.55 we also show *ϕ* for two different $\tilde{\kappa }$ at *ϵ* = 0.50. **(B)** Radial stress $\sigma_{rr}\left(r\right)$ for different bending rigidity $\tilde{\kappa }$ at *ϵ* = 0.50. Blue solid lines indicate a quadratic decay $\left(\sigma_{rr}\left(r\right)\propto r^{-2}\right)$ with the distance to the cell center, and black solid lines indicate $\left(\sigma_{rr}\left(r\right)\propto r^{-1}\right)$ .

Cell contraction leads to mechanical stresses in the surrounding network. To investigate how these stresses propagate for different contractile strains, we identify the nodes in the network that have at least one stretched or compressed bond. Here we define a bond to be stretched or compressed when the corresponding force *f*_*ij*_ is equal to or greater than a threshold $f^{\left(th\right)}$ , which we take to be the maximum localized force in the network when the energy exceeds the numerical error. Figure 2 shows snapshots of the stressed bonds in the network for four different connectivities, highlighting a dense, stressed region in the vicinity of the cell at high connectivity, which becomes more irregular in sparse networks with lower *p*. To investigate in more detail how the stress propagation depends on the cell contraction and network connectivity, we compute the fraction of nodes with at least one out-of-equilibrium bond as a function of the distance *r* to the cell centre, $\phi \left(r\right)$ :

$$\phi \left(r\right)=\left\langle \frac{N_{d}\left(r\right)}{N\left(r\right)}\right\rangle ,$$ (2)

where the brackets $\left\langle \cdot \right\rangle$ indicate ensemble averaging, $N_{d}\left(r\right)$ is the number of nodes at distance *r* that has a stretched or compressed bond (as specified above), and *N*(*r*) is the total number of bonds at distance *r*. These results are reported in Figure 3A for different strains *ϵ* and the four connectivities studied here at $\tilde{\kappa }=10^{-4}$ . For the highest connectivity, *p* = 0.85, we observe a region close to the cell where all bonds carry stress (i.e., *ϕ*(*r*) ≈ 1), which extends over larger distances as the strain increases. For larger distances, *ϕ*(*r*) decays, indicating that the stress pattern becomes more diffuse far away from the cell. The boundary between the dense and diffuse region is also indicated in the snapshot in Figure 2A. When the network connectivity decreases to *p* = 0.75, the fully stressed region shrinks and at *p* ≤ 0.65 it disappears completely. Remarkably, however, *ϕ*(*r*) develops a long tail, which decays over longer distances as the connectivity is reduced. This indicates that, while the stress pattern is more diffuse in sparsely connected networks, the mechanical perturbation can be perceived over greater distances as *p* decreases (Figure 3A). We also study how the bending rigidity $\tilde{\kappa }$ of the fibres influences the force propagation. For *p* ≥ 0.75, we do not find any significant difference in the behaviour of $\phi \left(r\right)$ (not shown for clarity). This is expected, because these networks are above the isostatic point, where the mechanical response is completely governed by fibre stretching (Broedersz et al., 2011). However, significant changes are observed at lower *p*, where bending modes become important. In particular, for *p* = 0.65 we see that increasing the bending rigidity leads to a higher fraction of stressed bonds close to the cell, while the decay at larger distances becomes steeper. From these results, we can conclude that stresses tend to concentrate in a region around the contractile cell in rigid fibre networks, while for sparser, softer networks, the stresses branch out over a large but very diffuse area.

We further characterize the propagation of forces generated by the cell by computing the local stress tensor at each bond connecting nodes *i* and *j* (Ronceray et al., 2016), defined as

$$\sigma_{\alpha \beta }^{\left(ij\right)}=-f_{\alpha }^{\left(ij\right)}\left[r_{\beta }^{\left(j\right)}-r_{\alpha }^{\left(i\right)}\right],$$ (3)

where $f_{\alpha }^{\left(ij\right)}$ is the *α*-component of the force supported by the bond between nodes *i* and *j*, and $r_{\beta }^{\left(j\right)}$ is the *β*-component of the position of bond *j*. In particular, we compute the radial component *σ*_*rr*_ and average this for each radial distance *r*, using circular bins of thickness Δ*r*. In Figure 3B we show $\sigma_{rr}\left(r\right)$ (averaged over many independent network realizations) as a function of $\tilde{\kappa }$ for all connectivities discussed here, at a strain *ϵ* = 0.50. For *p* = 0.85, we find that $\sigma_{rr}\left(r\right)\propto r^{-2}$ up to a distance *r* ≤ r*, which corresponds to the transition from the fully stressed inner region to the more diffuse outer region. This decay is consistent with the stress profile expected for continuous, linearly elastic media in two dimensions (Ronceray et al., 2016). For *p* = 0.75, the stress exhibits a more complex behaviour, emphasised by the presence of a slower decay as $\sigma_{rr}\left(r\right)\propto r^{-1}$ in the vicinity of the cell, to later recover the linearly elastic decay $\sigma_{rr}\left(r\right)\propto r^{-2}$ . As discussed previously (Ronceray et al., 2016), this cross-over is related to the non-linear response of the fibre network and, in particular, to collective buckling modes in the inner region, which prevents the network from sustaining compressive stresses in this region. We also see that for *p* ≥ 0.75, the bending rigidity does not influence the stress profile, in agreement with our observations for *ϕ*(*r*). However, for *p* ≤ 0.65, the bending rigidity does play an important role. Indeed, for *p* = 0.65 we find a transition from $\sigma_{rr}\left(r\right)\propto r^{-2}$ at high $\tilde{\kappa }$ (most rigid networks) to a slower decay $\sigma_{rr}\left(r\right)\propto r^{-1}$ at low $\tilde{\kappa }$ (softest networks), while for *p* = 0.55 the linear elasticity decay disappears completely and the stress decays as *r* ^−1^ for all $\tilde{\kappa }$ . This slow decay is related to the formation of so-called force chains, linear chains of stretched bonds that radiate outward. As we will see below, compressive stresses are irrelevant in this regime, while the tension in the force chains leads to a radial stress that decreases proportionally to the local density of force chains, which goes as 1/*r*.

### 3.2 Pattern of Force Transmission

**FIGURE 4:**
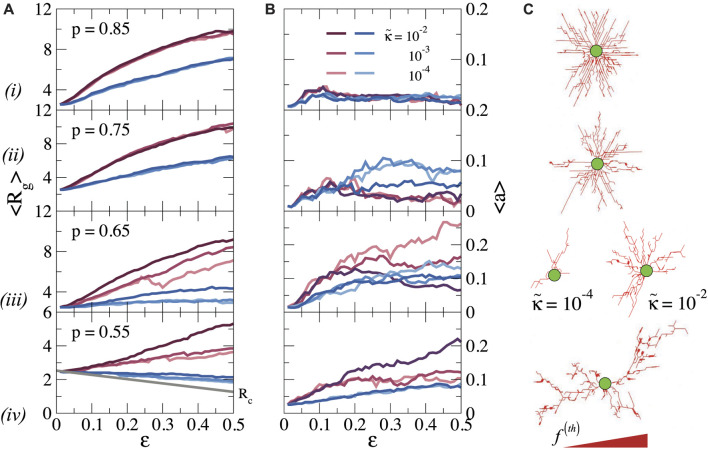
**(A)** Average radius of gyration *R*_*g*_, and **(B)** asphericity *a* as a function of *ϵ* and bending rigidity $\tilde{\kappa }$ for the cluster of compressed (blue) and stretched (red) bonds, and **(C)** the corresponding snapshots for stretched bonds at *ϵ* = 0.50 and $\tilde{\kappa }=10^{-4}$ for **(i)** *p* = 0.85, **(ii)** *p* = 0.75, **(iii)** *p* = 0.65 and **(iv)** *p* = 0.55. For *p* = 0.65, we also show a snapshot of stretched bonds for $\tilde{\kappa }=10^{-2}$ , highlighting that a more symmetric pattern can be recovered by increasing the bending rigidity. The bond thickness is proportional to force magnitude. Solid gray line in (a.iv) indicates the evolution of the cell radius *R*_*c*_ during contraction.

Next, we study the pattern of the forces in more detail. We treat compressed and stretched bonds separately, as shown in Supplementary Figures S1A,B. Likewise, we only consider nodes connected to the cell surface *via* other deformed bonds. For each resulting cluster of deformed bonds, we compute the gyration tensor as

$$S_{\alpha \beta }=\frac{1}{N_{G}}\overset{N_{G}}{\underset{i}{\sum }}r_{i,\alpha }r_{i,\beta },$$ (4)

where *r*_*i*,*α*_ is the *α* − coordinate of particle *i* and where the sum runs over all *N*_*G*_ nodes that are part of the deformed cluster. We diagonalize the tensor obtaining the principal moments, *λ*_1_ and *λ*_2_ and from this we compute the radius of gyration $R_{g}=\sqrt{\lambda_{1}+\lambda_{2}}$ and the asphericity $a=\frac{{\left(\lambda_{1}-\lambda_{2}\right)}^{2}}{{\left(\lambda_{1}+\lambda_{2}\right)}^{2}}$ , which takes values between 0 and 1 to indicate deviations from circular symmetry. The behaviour of *R*_*g*_ and *a* are shown as a function of strain in Figures 4A,B, respectively, for different values of $\tilde{\kappa }$ , while Figure 4C shows corresponding snapshots for the stretched bonds.

We first discuss the pattern of stretched bonds. For *p* ≥ 0.75 we find that *R*_*g*_ increases continuously with *ϵ*, preserving the spherical symmetry, as indicated by the low value of *a*. The growth of *R*_*g*_ with *ϵ* obviously is related to the growth of the stressed region seen in Figure 3A, and indicates how the stresses propagate further out as the cell contracts more. Again, the bending rigidity is unimportant in this stretching-dominated regime. When the connectivity is reduced to *p* = 0.65, we observe that *R*_*g*_ becomes dependent on $\tilde{\kappa }$ . In particular, for low $\tilde{\kappa }$ the growth of *R*_*g*_ as a function of strain becomes erratic, which is due to large buckling-type rearrangements of nodes. The pattern is also highly asymmetric in these cases, as indicated by the relatively large value of *a*. When $\tilde{\kappa }$ increases and the network rigidity increases, the erratic behaviour of *R*_*g*_ disappears and the pattern becomes more isotropic, similar to the patterns at higher connectivity. This is also illustrated by the snapshots in Figure 4C for *p* = 0.65 and two different $\tilde{\kappa }$ . For the lowest connectivity, *p* = 0.55 the pattern remains highly anisotropic for all values of the bending rigidity. For such diluted networks, we also observe large variations between different network configurations, so that the ensemble average shown in Figures 4A,B gives a somewhat distorted view. As shown in Supplementary Figure S2, for individual network realization *R*_*g*_ grows erratically, with significant jumps that mark a sudden transition from floppy to rigid structures locally. This erratic behaviour also leads to very large differences between different network realizations for *p* = 0.55, in particular for low values of $\tilde{\kappa }$ , which highlights that force transmission is less robust and reliable in sparse networks than in denser networks.

Repeating this analysis for the compressed bonds, we see that for all *p* values *R*_*g*_ is significantly smaller than for the stretched bonds; for *p* = 0.55, *R*_*g*_ even decreases with increasing strain, as the cell pulls the nodes inwards. Hence, compression forces do not propagate far away from the cell surface, especially for low *p* and the transmission of forces over long distances is dominated by stretched bonds.

We note, finally, that our previous observation that forces can propagate over longer distances in more sparsely connected networks (Figure 3) does not lead to a larger radius of gyration of the force patterns. This is because the stress propagation in the more distant regions for low connectivity is governed by a small number of force chains, which contribute less to *R*_*g*_ than the dense zone of stressed bonds at higher connectivity.

### 3.3 Force Chains in the Network

As is clear from the snapshots in Figures 2, 4C, the pattern of the local forces differs greatly between networks of high and low connectivity. While the forces radiate outwards more or less isotropically at high *p*, the pattern at lower *p* is characterized by so-called force chains, sequences of stretched bonds that can transmit forces over long distances in certain directions (Grill et al., 2021). To analyze the pattern of these force chains in more detail, we follow previous work that explored force transmission in granular systems using concepts of graph theory (Bassett et al., 2015; Newman, 2018). It is well-established that a granular material (i.e., a material consisting of jammed granular particles) can be mapped on an athermal network, with contact forces between neighbouring particles represented as bonds between nodes. The mechanical properties of such granular materials are characterized by discrete force chains that are very similar to the force chains observed in our simulations. Mechanical forces are transmitted along these force chains, while regions between the force chains are shielded from mechanical stresses (Tordesillas, 2007; Somfai et al., 2005; Owens and Daniels, 2011).

**FIGURE 5:**
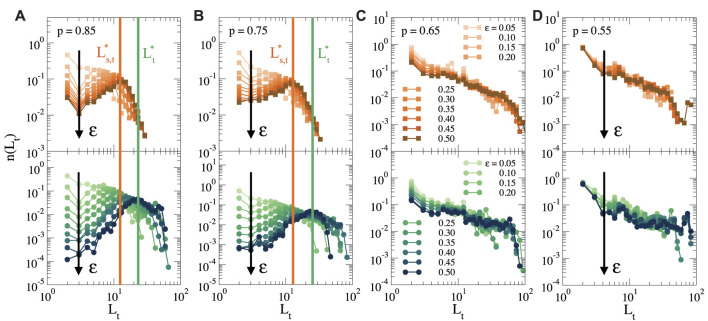
Topological length distribution of force chains that connect the periphery of the cluster to the cell surface for different strains (as indicated by the black arrow), and for **(A)** *p* = 0.85, **(B)** *p* = 0.75, **(C)** *p* = 0.65 and **(D)** *p* = 0.55. *Top:* Length distribution of the shortest paths, and *bottom:* length distribution of all simple paths. The vertical orange line indicates the characteristic shortest path $L_{s,t}^{*}$ and the vertical green line the characteristic mean path length $L_{t}^{*}$ . Here, $\tilde{\kappa }=10^{-4}$ .

To analyze the network of force chains, we start from the cluster of stretched bonds, as shown in Figure 4C, and we first identify the nodes at the end of each force chain, i.e., the nodes at the periphery of the cluster after which the force propagates no further (see also Supplementary Figure S1C). We then construct all simple paths, i.e., all non-repeating sequences of nodes (Newman, 2018), that connect each of the nodes at the periphery to the cell surface, obtaining thus a distribution of all force chains. In Figure 5 we show the distribution of the topological lengths of these force chains, for different strains and different connectivities. The top row shows only the shortest paths between each periphery node and the cell surface, as a measure for the typical distance over which the force propagates, while the bottom row shows the distribution of all simple paths. A difference between the distribution of shortest paths and all paths indicates the presence of many secondary paths due to cross-connections between force chains. Such cross-connections make the propagation of forces more robust, since the mechanical transmission does not rely on one single path, but multiple paths can transmit the force.

For *p* = 0.85 (Figure 5A) we see that at low strains, the distribution of path lengths decays monotonically, indicating that most of the force chains are short. However, when *ϵ* increases, the distribution acquires a clear optimum. This reveals that there is a characteristic length $L_{s,t}^{*}$ over which forces propagate. This characteristic length is in agreement with our previous observation that the force pattern is rather isotropic for this connectivity (Figure 4), so that force chains reach the same distance in any direction. As the strain induced by the contracting cell increases, the characteristic length for force propagation increases, until at larger strains (*ϵ* → 0.5) it appears to saturate (see also Supplementary Figure S3A). The distribution for all simple paths follows a similar trend as that for the shortest paths, but the maximum in the distribution, $L_{t}^{*}$ lies at larger lengths. With increasing strain the two distributions start to deviate more (see also Supplementary Figure S3A), indicating that there are many interconnected force paths, in agreement with the formation of a dense, fully stressed region near the cell shown in Figures 2, 3. For *p* = 0.75 (Figure 5) we find a similar behaviour, although the peaks associated with the characteristic lengths $L_{s,t}^{*}$ and $L_{t}^{*}$ are wider, indicating that for lower connectivity there is a larger variation in the typical distance for force transmission. For *p* ≤ 0.65, the situation is completely different. As shown in Figures 5C,D, for both *p* = 0.65 and *p* = 0.55, the length distribution decays monotonically for all strains, and follows a power law decay $n\left(L_{t}\right)\propto L_{t}^{-\alpha }$ with *α* ≈ 1 over a large range of lengths. Hence, there is no characteristic length of force propagation in these dilute networks. Most force chains are very short, but a small number of force chains can reach out far. Again, this is in agreement with the large apshericity and the anisotropic force patterns shown in Figure 4. For these low connectivities, we also find that the distribution of all simple paths is nearly the same as that of only the shortest paths (Supplementary Figure S3B), indicating that there are few interconnections between force chains, so that long-range force transmission relies on one or a few force chains only. We also explore the influence that $\tilde{\kappa }$ has on the force chains. As expected, for *p* ≥ 0.75 the bending rigidity does not modify the path length distributions, but for *p* = 0.65 an increase in bending rigidity promotes a characteristic distance, making the force chain network more similar to that for higher connectivities (see Supplementary Figure S3A).

**FIGURE 6:**
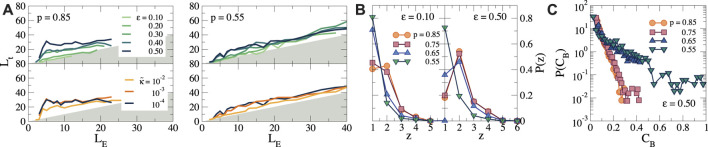
**(A)** The topological length *L*_*t*_ of the force chains as a function of the Euclidean distance that the force chain reaches *L*_*E*_, for different strains *ϵ* and bending rigidity $\tilde{\kappa }=10^{-4}$ (top), and *ϵ* = 0.50 and different $\tilde{\kappa }$ (bottom) for *p* = 0.85 and *p* = 0.55. **(B)** Connectivity distribution $P\left(z\right)$ of nodes in the force network as a function of *p* for *ϵ* = 0.10 and 0.50. **(C)** Probability distribution function of the node betweenness centrality $P\left(C_{B}\right)$ for different *p* and for *ϵ* = 0.50 in semi-log scale. In **(B,C)**, $\tilde{\kappa }=10^{-4}$ .

To analyze the morphology of the force chain network in more detail, we plot the topological length of each force chain in the network *L*_*t*_ as a function of the Euclidean distance *L*_*E*_ between the end of the force chain and the cell surface, see Figure 6 for *p* = 0.85 and 0.55, and Supplementary Figure S4 for *p* = 0.75 and 0.65. Here, *L*_*t*_ = *L*_*E*_ corresponds to a straight force chain, while *L*_*t*_ > *L*_*E*_ corresponds to a curved or irregular force chain Ref. (Bassett et al., 2015) (note that *L*_*t*_ cannot be smaller than *L*_*E*_, so that the grey area in Figure 6 is unphysical). For small strains (*ϵ* ≤ 0.10), we observe that *L*_*t*_ ≃ *L*_*E*_ for all *p*. However, as *ϵ* increases, we find significant deviations from straight force chains for *p* = 0.85 and 0.75 (see Figure 6A and Supplementary Figure S3B, respectively), especially in the dense inner region, due to alternative paths that link the periphery of the force network to the cell surface. By contrast, for *p* = 0.65 and 0.55 and for larger $\tilde{\kappa }$ , the difference between *L*_*t*_ and *L*_*E*_ remains smaller as a result of the reduced number of interconnections between force chains, indicating that most force chains follow a more or less straight path outward.

The complex structure of all simple force paths that emerges can be further analyzed by computing the distribution degree $P\left(z\right)$ of the nodes in the cluster of stretched bonds, where *z* indicates the number of stretched bonds connected to a node in the cluster: *z* = 1 corresponds to the end nodes of the force chains, *z* = 2 denotes linear sections of the force chains, and *z* ≥ 3 represents branches. In Figure 6B, we show $P\left(z\right)$ for different *p* and for *ϵ* = 0.10 and 0.50. For high connectivities, we observe that *P*(*z*) develops a peak at *z* = 2 as the strain increases, with a significant number of nodes with *z* ≥ 3, as expected for a network of highly interconnected force chains. As the connectivity decreases, the number of such interconnections decreases as well. For *p* = 0.55, *P*(*z*) decreases monotonically with *z*, implying that most of the force chains extend only over one bond in this case, with only a few longer force chains. The number of nodes with *z* ≥ 3 is very small for this connectivity, indicating few branches and interconnections between force chains.

Another way to characterize the force transmission in the networks is by analyzing the node betweenness centrality *C*_*B*_ for each node in the network. This is a measure often used in graph theory to denote the importance of a certain node for transmission within a network, and is defined as (Brandes, 2001):

$$C_{B}\left(j\right)=\underset{i,k\in N_{G}}{\sum }\frac{n_{i,k}^{j}}{n_{i,k}}$$ (5)

where *n*_*i*,*k*_ is the number of shortest paths between nodes *i* and *k*, and where $n_{i,k}^{j}$ is the number of these paths that goes through node *j*. Nodes with a high *C*_*B*_ are crossed by many shortest paths, which indicates that they have a greater influence on the transmission of forces. We show the distribution of the betweenness centrality $P\left(C_{B}\right)$ for the different *p* and for *ϵ* = 0.50 in Figure 6C. For the highest connectivities, there are almost no nodes with a high *C*_*B*_, because there are many shortest paths in the highly connected network of force chains. For *p* ≤ 0.65, however, the fraction of nodes with a high *C*_*B*_ is much larger, as indicated by the long tail in the distribution. In these networks, force transmission occurs by long linear force chains, where all bonds in the chain are essential for ensuring proper force propagation. Clearly, the removal of one node in the network for *p* ≤ 0.65 has a much more dramatic effect on the propagation of forces than at higher connectivities.

## 4 Concluding Remarks

Mechanical communication between cells relies on force transmission over large distances through the extracellular matrix (Janmey and Miller, 2011). The disordered structure of the matrix, its large mesh size and its mechanical non-linearity make this a highly non-trivial process. Our results highlight how the connectivity of the fibre network and the bending rigidity of the collagen fibrils influence the local force transmission. On the one hand, for highly connected (and therefore relatively stiff) networks, the forces propagate isotropically in all directions over a characteristic distance that can be controlled by the contraction of the cell. On the other hand, in dilute (and soft) networks, forces propagate along a few force chains that can transmit forces over very long distances, but only in a few directions. This communication is less reliable and robust than for more rigid networks, which may be one of the reasons for the large variability and heterogeneity in cell behaviour in such matrices. In particular, networks close to the rigidity threshold (*p* = 0.65 in our case) are very sensitive to bending rigidity. These findings may be relevant for cell and tissue morphology and collective cell migration in environments of different rigidity. Indeed, our results are consistent with various experimental studies, which have reported that the distance over which cells can communicate mechanically appears to depend on the stiffness of the matrix (Guo et al., 2006; Reinhart-King et al., 2008; Winer et al., 2009; Janmey and Miller, 2011; Koorman et al., 2022). Furthermore, we speculate that the appearance of a characteristic transmission length that emerges around the cell when local stiffness increases may be related to the observation that cells in a colony organise at a typical distance from each other (Reinhardt-King et al., 2008).

We hope that this paper will provide an incentive for future research on force transmission in disordered networks, as many questions remain open. For example, we have considered only uniform cell contraction, but previous work has suggested that cells often contract anisotropically to influence the direction of stress propagation (Baker and Chen, 2012; Koch et al., 2012; Ahmadzadeh et al., 2017). Furthermore, we have here used a rigid contractile body to model the cell. It would be interesting to study how mechanical feedback between the matrix and the cell emerges when the cell is itself modelled as a soft deformable object, for example, by treating the perimeter of the cell as a ring of springs that can stretch and bend (Gandikota et al., 2020). In such cases the anisotropic force chains may lead to spontaneous polarization of the cell. Cells can also actively restructure the matrix around them by inducing plastic deformations and thus influencing the propagation of mechanical signals (Vader et al., 2009; Kim et al., 2017). In addition, mechanical signalling may be affected by the hydrodynamic coupling between the collagen fibrils and the embedding fluid (Yucht et al., 2013; Head and Storm, 2019), as well as by the complex network composed of polysaccharides and glycosylated proteins in which collagen fibrils are embedded (Mouw et al., 2014; Burla et al., 2019b). Finally, we emphasise that the networks that we have studied here are 2D. While such networks have been shown previously to be excellently suited for characterising the mechanical properties of experimental collagen networks experimentally (Sharma et al., 2016a; Sharma et al., 2016b), it would be interesting to observe how force transmission occurs in 3D networks, introducing thus an additional degree of freedom to relax the local deformation in the network.

Finally, we suggest the possibility of using graph theory to characterize the mechanical propagation and the local distortion generated by cells on the ECM. In addition to the characteristics used here, many additional descriptors can be used to characterize the network’s topology, also experimentally. These parameters may be used to train neural networks, for example, to develop a machine learning-based approach to identify cell-matrix and cell-cell interactions. This could eventually be used as a diagnostic tool or help to design synthetic matrices with optimal mechanical characteristics for mechanical feedback.

## Data Availability

The original contributions presented in the study are included in the article/Supplementary Material, further inquiries can be directed to the corresponding authors.

## References

[B1] Ahmadzadeh H. Webster M. R. Behera R. Jimenez Valencia A. M. Wirtz D. Weeraratna A. T. (2017). Modeling the Two-Way Feedback Between Contractility and Matrix Realignment Reveals a Nonlinear Mode of Cancer Cell Invasion. Proc. Natl. Acad. Sci. U. S. A. 114, E1617–E1626. 10.1073/pnas.1617037114 28196892 PMC5338523

[B2] Arzash S. Shivers J. L. MacKintosh F. C. (2020). Finite Size Effects in Critical Fiber Networks. Soft Matter 16, 6784–6793. 10.1039/d0sm00764a 32638813

[B3] Baker B. M. Chen C. S. (2012). Deconstructing the Third Dimension: How 3D Culture Microenvironments Alter Cellular Cues. J. Cell Sci. 125, 3015–3024. 10.1242/jcs.079509 22797912 PMC3434846

[B4] Bassett D. S. Owens E. T. Porter M. A. Manning M. L. Daniels K. E. (2015). Extraction of Force-Chain Network Architecture in Granular Materials Using Community Detection. Soft Matter 11, 2731–2744. 10.1039/c4sm01821d 25703651

[B5] Bates J. H. T. Davis G. S. Majumdar A. Butnor K. J. Suki B. (2007). Linking Parenchymal Disease Progression to Changes in Lung Mechanical Function by Percolation. Am. J. Respir. Crit. Care Med. 176, 617–623. 10.1164/rccm.200611-1739oc 17575096 PMC1994222

[B6] Bitzek E. Koskinen P. Gähler F. Moseler M. Gumbsch P. (2006). Structural Relaxation Made Simple. Phys. Rev. Lett. 97, 170201. 10.1103/physrevlett.97.170201 17155444

[B7] Brandes U. (2001). A Faster Algorithm for Betweenness Centrality*. J. Math. Sociol. 25, 163–177. 10.1080/0022250x.2001.9990249

[B8] Broedersz C. P. MacKintosh F. C. (2014). Modeling Semiflexible Polymer Networks. Rev. Mod. Phys. 86, 995–1036. 10.1103/revmodphys.86.995

[B9] Broedersz C. P. Mao X. Lubensky T. C. MacKintosh F. C. (2011). Criticality and Isostaticity in Fibre Networks. Nat. Phys. 7, 983–988. 10.1038/nphys2127

[B10] Burla F. Dussi S. Martinez-Torres C. Tauber J. van der Gucht J. Koenderink G. H. (2020). Connectivity and Plasticity Determine Collagen Network Fracture. Proc. Natl. Acad. Sci. U.S.A. 117, 8326–8334. 10.1073/pnas.1920062117 32238564 PMC7165426

[B11] Burla F. Mulla Y. Vos B. E. Aufderhorst-Roberts A. Koenderink G. H. (2019a). From Mechanical Resilience to Active Material Properties in Biopolymer Networks. Nat. Rev. Phys. 1, 249–263. 10.1038/s42254-019-0036-4

[B12] Burla F. Tauber J. Dussi S. van Der Gucht J. Koenderink G. H. (2019b). Stress Management in Composite Biopolymer Networks. Nat. Phys. 15, 549–553. 10.1038/s41567-019-0443-6

[B13] Causa F. Netti P. A. Ambrosio L. (2007). A Multi-Functional Scaffold for Tissue Regeneration: the Need to Engineer a Tissue Analogue. Biomaterials 28, 5093–5099. 10.1016/j.biomaterials.2007.07.030 17675151

[B14] Chen C. S. Tan J. Tien J. (2004). Mechanotransduction at Cell-Matrix and Cell-Cell Contacts. Annu. Rev. Biomed. Eng. 6, 275–302. 10.1146/annurev.bioeng.6.040803.140040 15255771

[B15] DuChez B. J. Doyle A. D. Dimitriadis E. K. Yamada K. M. (2019). Durotaxis by Human Cancer Cells. Biophysical J. 116, 670–683. 10.1016/j.bpj.2019.01.009 PMC6382956 30709621

[B16] Gandikota M. C. Pogoda K. Van Oosten A. Engstrom T. A. Patteson A. E. Janmey P. A. (2020). Loops versus Lines and the Compression Stiffening of Cells. Soft matter 16, 4389–4406. 10.1039/c9sm01627a 32249282 PMC7225031

[B17] Goren S. Koren Y. Xu X. Lesman A. (2020). Elastic Anisotropy Governs the Range of Cell-Induced Displacements. Biophysical J. 118, 1152–1164. 10.1016/j.bpj.2019.12.033 PMC7063433 31995739

[B18] Grill M. J. Kernes J. Slepukhin V. M. Wall W. A. Levine A. J. (2021). Directed Force Propagation in Semiflexible Networks. Soft Matter 17, 10223–10241. 10.1039/d0sm01177k 33367438

[B19] Guo W.-h. Frey M. T. Burnham N. A. Wang Y.-l. (2006). Substrate Rigidity Regulates the Formation and Maintenance of Tissues. Biophysical J. 90, 2213–2220. 10.1529/biophysj.105.070144 PMC1386800 16387786

[B20] Han Y. L. Ronceray P. Xu G. Malandrino A. Kamm R. D. Lenz M. (2018). Cell Contraction Induces Long-Ranged Stress Stiffening in the Extracellular Matrix. Proc. Natl. Acad. Sci. U.S.A. 115, 4075–4080. 10.1073/pnas.1722619115 29618614 PMC5910866

[B21] Head D. Storm C. (2019). Nonaffinity and Fluid-Coupled Viscoelastic Plateau for Immersed Fiber Networks. Phys. Rev. Lett. 123, 238005. 10.1103/physrevlett.123.238005 31868451

[B22] Hinz B. Phan S. H. Thannickal V. J. Prunotto M. Desmoulière A. Varga J. (2012). Recent Developments in Myofibroblast Biology: Paradigms for Connective Tissue Remodeling. Am. J. pathology 180, 1340–1355. 10.1016/j.ajpath.2012.02.004 PMC3640252 22387320

[B23] Janmey P. A. Miller R. T. (2011). Mechanisms of Mechanical Signaling in Development and Disease. J. cell Sci. 124, 9–18. 10.1242/jcs.071001 21172819 PMC3001405

[B24] Jansen K. A. Licup A. J. Sharma A. Rens R. MacKintosh F. C. Koenderink G. H. (2018). The Role of Network Architecture in Collagen Mechanics. Biophysical J. 114, 2665–2678. 10.1016/j.bpj.2018.04.043 PMC6129505 29874616

[B25] Jones C. A. Cibula M. Feng J. Krnacik E. A. McIntyre D. H. Levine H. (2015). Micromechanics of Cellularized Biopolymer Networks. Proc. Natl. Acad. Sci. U. S. A. 112, E5117–E5122. 10.1073/pnas.1509663112 26324923 PMC4577196

[B26] Kim J. Feng J. Jones C. A. R. Mao X. Sander L. M. Levine H. (2017). Stress-induced Plasticity of Dynamic Collagen Networks. Nat. Commun. 8, 842–847. 10.1038/s41467-017-01011-7 29018207 PMC5635002

[B27] Koch T. M. Münster S. Bonakdar N. Butler J. P. Fabry B. (2012). 3d Traction Forces in Cancer Cell Invasion. Plos one 7, e33476. 10.1371/journal.pone.0033476 22479403 PMC3316584

[B28] Koorman T. Jansen K. A. Khalil A. Haughton P. D. Visser D. Rätze M. A. (2022). Spatial Collagen Stiffening Promotes Collective Breast Cancer Cell Invasion by Reinforcing Extracellular Matrix Alignment. Oncogene 43, 1–12. 10.1038/s41388-022-02258-1 PMC9033577 35292774

[B29] Lecuit T. Lenne P.-F. Munro E. (2011). Force Generation, Transmission, and Integration during Cell and Tissue Morphogenesis. Annu. Rev. Cell Dev. Biol. 27, 157–184. 10.1146/annurev-cellbio-100109-104027 21740231

[B30] Li B. Wang J. H.-C. (2011). Fibroblasts and Myofibroblasts in Wound Healing: Force Generation and Measurement. J. tissue viability 20, 108–120. 10.1016/j.jtv.2009.11.004 19995679 PMC2891362

[B31] Licup A. J. Münster S. Sharma A. Sheinman M. Jawerth L. M. Fabry B. (2015). Stress Controls the Mechanics of Collagen Networks. Proc. Natl. Acad. Sci. U.S.A. 112, 9573–9578. 10.1073/pnas.1504258112 26195769 PMC4534289

[B32] Lo C.-M. Wang H.-B. Dembo M. Wang Y.-l. (2000). Cell Movement Is Guided by the Rigidity of the Substrate. Biophysical J. 79, 144–152. 10.1016/s0006-3495(00)76279-5 PMC1300921 10866943

[B33] Maxwell J. C. (1864). L. On the Calculation of the Equilibrium and Stiffness of Frames. Lond. Edinb. Dublin Philosophical Mag. J. Sci. 27, 294–299. 10.1080/14786446408643668

[B34] Mouw J. K. Ou G. Weaver V. M. (2014). Extracellular Matrix Assembly: a Multiscale Deconstruction. Nat. Rev. Mol. Cell Biol. 15, 771–785. 10.1038/nrm3902 25370693 PMC4682873

[B35] Narmoneva D. A. Wang J. Y. Setton L. A. (1999). Nonuniform Swelling-Induced Residual Strains in Articular Cartilage. J. Biomechanics 32, 401–408. 10.1016/s0021-9290(98)00184-5 10213030

[B36] Newman M. (2018). Networks. Oxford, United Kingdom: Oxford University Press.

[B37] Notbohm J. Lesman A. Rosakis P. Tirrell D. A. Ravichandran G. (2015). Microbuckling of Fibrin Provides a Mechanism for Cell Mechanosensing. J. R. Soc. Interface. 12, 20150320. 10.1098/rsif.2015.0320 26040601 PMC4528600

[B38] Owens E. T. Daniels K. E. (2011). Sound Propagation and Force Chains in Granular Materials. Epl Europhys. Lett. 94, 54005. 10.1209/0295-5075/94/54005

[B39] Reinhart-King C. A. Dembo M. Hammer D. A. (2008). Cell-cell Mechanical Communication through Compliant Substrates. Biophysical J. 95, 6044–6051. 10.1529/biophysj.107.127662 PMC2599854 18775964

[B40] Rens E. G. Merks R. M. H. (2020). Cell Shape and Durotaxis Explained from Cell-Extracellular Matrix Forces and Focal Adhesion Dynamics. Iscience 23, 101488. 10.1016/j.isci.2020.101488 32896767 PMC7482025

[B41] Rens R. Vahabi M. Licup A. J. MacKintosh F. C. Sharma A. (2016). Nonlinear Mechanics of Athermal Branched Biopolymer Networks. J. Phys. Chem. B 120, 5831–5841. 10.1021/acs.jpcb.6b00259 26901575

[B42] Ronceray P. Broedersz C. P. Lenz M. (2016). Fiber Networks Amplify Active Stress. Proc. Natl. Acad. Sci. U.S.A. 113, 2827–2832. 10.1073/pnas.1514208113 26921325 PMC4801261

[B43] Sharma A. Licup A. J. Jansen K. A. Rens R. Sheinman M. Koenderink G. H. (2016a). Strain-controlled Criticality Governs the Nonlinear Mechanics of Fibre Networks. Nat. Phys. 12, 584–587. 10.1038/nphys3628

[B44] Sharma A. Licup A. J. Rens R. Vahabi M. Jansen K. A. Koenderink G. H. (2016b). Strain-driven Criticality Underlies Nonlinear Mechanics of Fibrous Networks. Phys. Rev. E 94, 042407. 10.1103/PhysRevE.94.042407 27841637

[B45] Somfai E. Roux J. N. Snoeijer J. H. Van Hecke M. Van Saarloos W. (2005). Elastic Wave Propagation in Confined Granular Systems. Phys. Rev. E Stat. Nonlin Soft Matter Phys. 72, 021301. 10.1103/PhysRevE.72.021301 16196550

[B46] Sopher R. S. Tokash H. Natan S. Sharabi M. Shelah O. Tchaicheeyan O. (2018). Nonlinear Elasticity of the Ecm Fibers Facilitates Efficient Intercellular Communication. Biophysical J. 115, 1357–1370. 10.1016/j.bpj.2018.07.036 PMC6170818 30217380

[B47] Tauber J. Van Der Gucht J. Dussi S. (2022). Stretchy and Disordered: Toward Understanding Fracture in Soft Network Materials *via* Mesoscopic Computer Simulations. J. Chem. Phys. 156, 160901. 10.1063/5.0081316 35490006

[B48] Tordesillas A. (2007). Force Chain Buckling, Unjamming Transitions and Shear Banding in Dense Granular Assemblies. Philos. Mag. 87, 4987–5016. 10.1080/14786430701594848

[B49] Totsukawa G. Wu Y. Sasaki Y. Hartshorne D. J. Yamakita Y. Yamashiro S. (2004). Distinct Roles of Mlck and Rock in the Regulation of Membrane Protrusions and Focal Adhesion Dynamics during Cell Migration of Fibroblasts. J. cell Biol. 164, 427–439. 10.1083/jcb.200306172 14757754 PMC2172229

[B50] Vader D. Kabla A. Weitz D. Mahadevan L. (2009). Strain-induced Alignment in Collagen Gels. Plos One 4, e5902. 10.1371/journal.pone.0005902 19529768 PMC2691583

[B51] Wegst U. G. K. Bai H. Saiz E. Tomsia A. P. Ritchie R. O. (2015). Bioinspired Structural Materials. Nat. Mater 14, 23–36. 10.1038/nmat4089 25344782

[B52] Winer J. P. Oake S. Janmey P. A. (2009). Non-linear Elasticity of Extracellular Matrices Enables Contractile Cells to Communicate Local Position and Orientation. Plos One 4, e6382. 10.1371/journal.pone.0006382 19629190 PMC2711623

[B53] Yucht M. G. Sheinman M. Broedersz C. P. (2013). Dynamical Behavior of Disordered Spring Networks. Soft Matter 9, 7000–7006. 10.1039/c3sm50177a

