# Supplementary material for: Force Transmission in Disordered Fibre Networks
Source: Front Cell Dev Biol. 2022 Jun 30;10:931776. doi: 10.3389/fcell.2022.931776 (PMC9280074; doi:10.3389/fcell.2022.931776)
Supplement: Supplementary file 1 [file DataSheet1.PDF]

Supplementary Material

1 ADDITIONAL RESULTS

1.1 Compressed vs stretched bond patterns

Fig. S1 shows sub-networks considering only compressed or stretched bonds at $\tilde{\kappa} = 10^{-4}$ for $p = 0.85$ and $p = 0.55$. These sub-networks are analyzed by removing disconnected nodes in order to define a cluster, as we represent in Fig. S1(c) for the stretched bonds, and we compute the radius of gyration R_g and the asphericity a. Furthermore, we compute the shortest and all simple paths linking the cell with the nodes at the periphery, defining a kind of surface, represented in Fig. S1(c) by the black dots.

1.2 Radius of gyration of different samples

In Fig. S2 we show the radius of gyration R_g of the stressed cluster for three independent samples at $p = 0.85$ and 0.55 as a function of ϵ for $\tilde{\kappa} = 10^{-4}$. We can observe a steep erratic behavior of R_g at low connectivities. This erratic behavior corresponds to a local transition from soft to rigid state. Likewise, some samples show higher R_g at $p = 0.55$. This result highlights the relevance of local density when the cell begins to transmit local forces.

1.3 Topology and morphology of the simple pathway

In Fig. S3(a) we report the evolution of the characteristic topological length for the shortest $L^*_{s,t}$ and all simple paths L^*_t as a function of ϵ, for $p = 0.85$ and 0.75. In addition, Fig. S3(b) shows the topological length distribution of force chains at $\epsilon = 0.50$ for both types of paths, as a function of p, indicating that for $p \leq 0.65$ the distribution of both type of paths are quite similar.

In Fig. S4(a) we report $n\left(L_t\right)$ for $p = 0.65$ and $p = 0.55$ as a function of $\tilde{\kappa}$. Fig. S4(b) and (c) show the morphology of the simple paths as a function of ϵ for $p = 0.75$ and 0.65. As discussed in the main text, $L_t = L_E$ is the case of a perfect chain, while $L_t > L_E$ corresponds to the presence of complex structures such as branches or gaps. Finally, the shaded region corresponds to $L_t < L_E$.

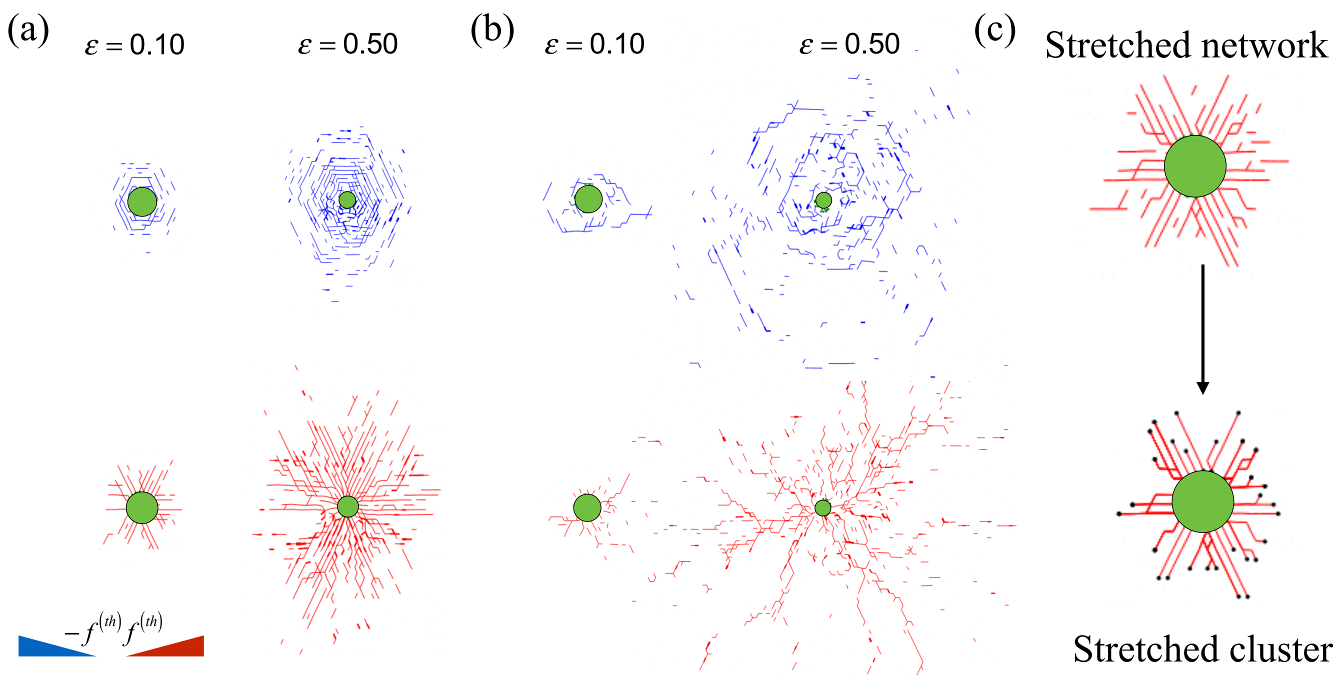

Figure S1. Snapshots showing the compressed (bonds with $f_{ij} \leq -f^{(th)}$) and stretched (bonds with $f_{ij} \geq f^{(th)}$) sub-networks, as function of the local compression induced by the intruder with (a) $p = 0.85$ and (b) $p = 0.55$. Bond width corresponds to the force magnitude. (c) Definition of a cluster with stretched bonds, where black dots highlight the periphery nodes considered to study the simple paths.

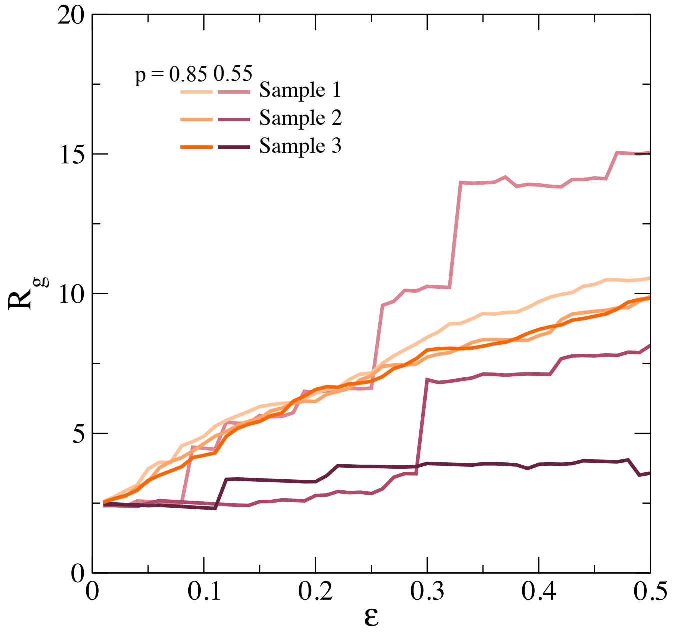

Figure S2. Radius of gyration R_g of the cluster with stretched bonds for three different samples at $p = 0.85$ and 0.55 as a function of the strain ϵ for $\tilde{\kappa} = 10^{-4}$

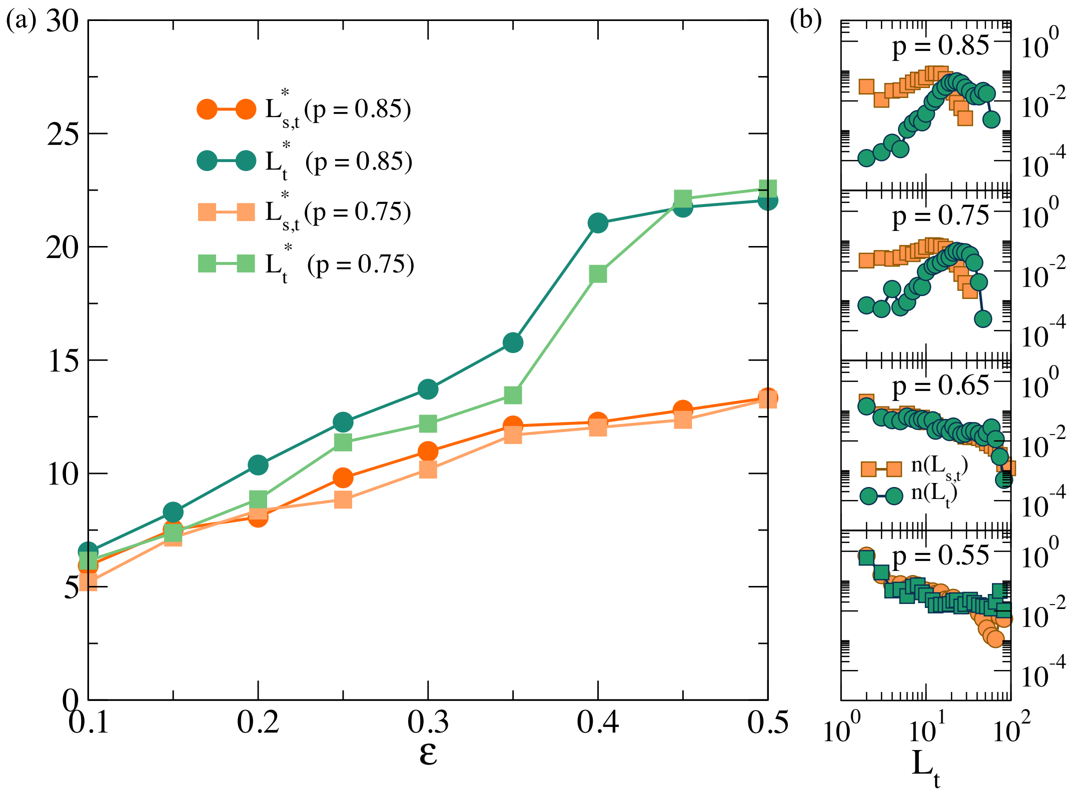

Figure S3. (a) Characteristic topological length for shortest $L^*_{s,t}$ and simple L^*_t paths as a function of the strain ϵ for $p = 0.85$ and 0.75. (b) Topological length distribution of the shortest paths (orange) and all simple paths (green) for strain $\epsilon = 0.50$ Here, $\tilde{\kappa} = 10^{-4}$.

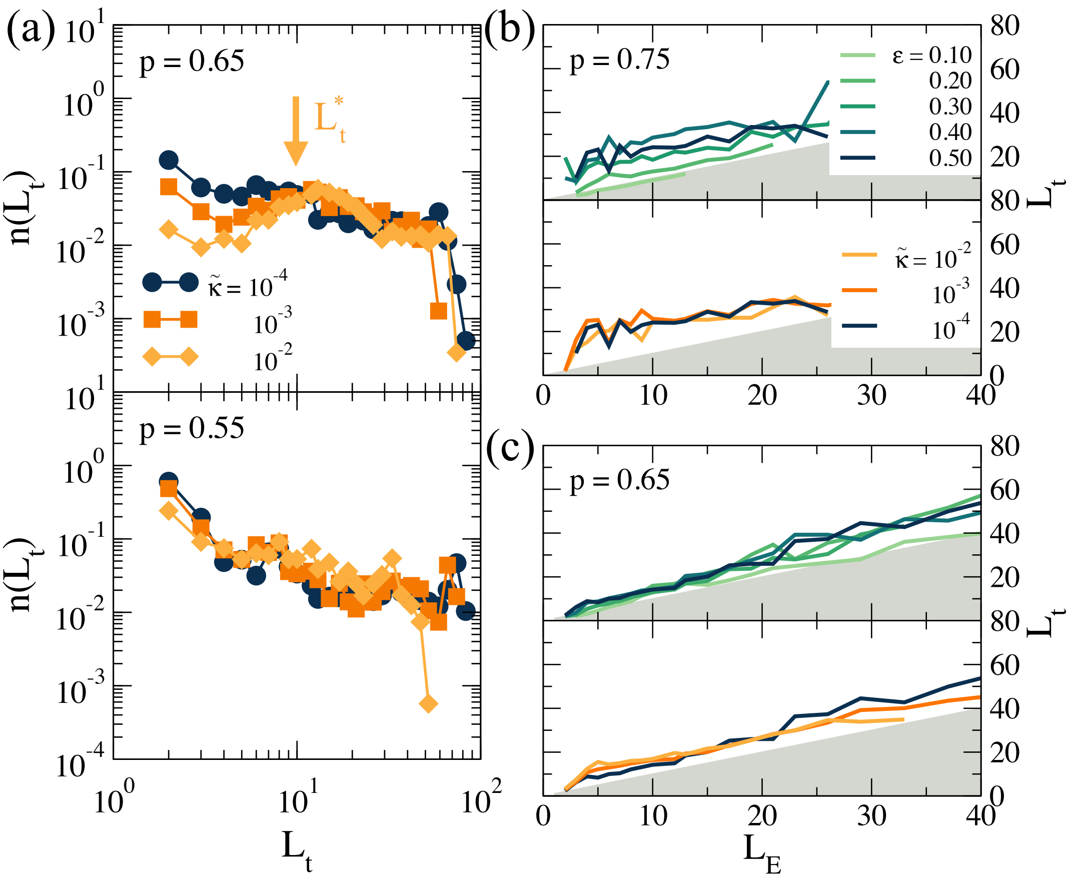

Figure S4. (a) Topological length distribution of all simple paths that connect the periphery of the cluster to the cell surface for $\epsilon = 0.50$ and different $\tilde{\kappa}$, for $p = 0.65$ and 0.55. The orange arrow indicates the characteristic mean path length L_t^*. (b) The topological length L_t of the force chains as a function of the Euclidean distance that the force chain reaches L_E, for different strains ϵ and bending rigidity $\tilde{\kappa} = 10^{-4}$ (top), and $\epsilon = 0.50$ and different $\tilde{\kappa}$ (bottom) for $p = 0.75$ and $p = 0.65$.
